# Supplementary material for: Poorer Physical Function Is Associated With Elevated Spatial Entropy in the Aging Brain Network Landscape
Source: Aging Cell. 2026 Jun 18;25(6):e70596. doi: 10.1111/acel.70596 (PMC13277748; doi:10.1111/acel.70596)
Supplement: Supplementary file 1 — Table S1. Variable differences between younger and older adults. Figure S1. Spearman correlation matrix for key variables in the cohort of older adults. Red indicates positive correlation while blue indicates negative correlation. Figure S2. Results from replication analyses controlling for the number of volumes removed due to motion. Figure S3. Unthresholded map of T‐values from a regression of the interaction between age group and BMI against brain entropy maps. Figure S4. Unthresholded maps of T‐values from a regression of age against brain entropy maps in the cohort of older adults. Figure S5. Thresholded and unthresholded maps of T‐values from a two‐sample t‐test comparing entropy maps of older adults with and without diabetes history. Figure S6. Thresholded and unthresholded maps of T‐values from a regression of fasting blood glucose against brain entropy maps in the cohort of older adults. Figure S7. Thresholded and unthresholded maps of T‐values from a two‐sample t‐test comparing entropy maps of older adults with and without hypertension history. Figure S8. Thresholded and unthresholded maps of T‐values from a regression of systolic blood pressure against brain entropy maps in the cohort of older adults. Figure S9. Results from replication analyses controlling for fasting glucose and systolic blood pressure. Figure S10. Thresholded and unthresholded maps of T‐values from a two‐sample t‐test comparing entropy maps of older adults who do meet physical activity guidelines versus older adults who do not. Figure S11. Results from replication analyses using communities identified with the Leiden algorithm. Figure S12. Results from analyses of the relationship between gray matter volume and entropy and the effect of including gray matter volume as a covariate in regressions from the main analyses of this paper. [file ACEL-25-e70596-s002.docx]

**Extended Methods**

*Additional Health and Behavioral Measures*

All additional health measures are based on assessments performed at the participants’ baseline visits. Diabetes was defined as anyone that is currently taking diabetes medication or that had fasting serum glucose > 130 mg/dL. Hypertension was defined as anyone with systolic blood pressure > 150 mmHg or anyone taking hypertension medication with systolic blood pressure > 130 mmHg. Physical activity was assessed using responses to the Community Healthy Activities Model Program for Seniors (CHAMPS) questionnaire (Stewart et al., 2001). Responses were binarized such that physical activity was coded as “1” if the participant reported engaging in at least 150 minutes of moderate physical activity per week and “0” if they did not.

*Community Detection Sensitivity Analyses*

To assess whether findings were sensitive to the specific community detection algorithm used to identify communities in brain networks, we used the Leiden algorithm to identify communities from each participant’s brain network. These communities were then mapped to the brain and used to generate entropy maps. These entropy maps were used to replicate analyses presented in the main text that used communities identified with the Louvain stability algorithm.

*Gray Matter Volume Analyses*

We wanted to assess whether findings were driven by a higher degree of gray matter atrophy in the older sample compared or the younger sample, or whether differences in gray matter volume underly the relationships observed between entropy and eSPPB or BMI. Modulated gray matter maps calculated with ANTs were smoothed using a 10mm FWHM kernel and resliced from 1mm x 1mm x 1mm to 4mm x 4mm x 5mm to match the voxel dimensions of the entropy maps. The association between gray matter volume and entropy maps were assessed by fitting a linear regression to each voxel within the brain mask with entropy as the dependent outcome and gray matter volume as the independent outcome (both measures varied by person and by voxel). In additional analyses, the main findings from Figures 2-4 of the main paper were replicated while including the gray matter volume of each voxel as a covariate in regression models. Cluster correction was not completed for these particular analyses because regressions required a different value for gray matter volume to be entered for each voxel of each participant. To our knowledge, SPM does not support this type of analysis. However, we invite the reader to compare the unthresholded map of T-values from these analyses to the analogous maps in the main paper (Figures 2-4).

**Extended Results**

*Supplementary Table 1. Variable Differences between Younger and Older Adults*

| Variables | Older Adults (n = 192*) | Younger Adults (n = 30) | p-value |
| --- | --- | --- | --- |
| Age (years) | 76.4±4.7 | 30.0±3.8 | **<0.0001** |
| Expanded Short Physical Performance Battery | 2.0±0.5 | 2.8±0.2 | **<0.0001** |
| Body Mass Index (kg/m^2^) | 28.4±5.5 | 25.5±5.1 | **0.0071** |
| Systolic Blood Pressure (mmHg) | 138.7±17.9 | 118.2±17.3 | **<0.0001** |
| Fasting Glucose (mg/dL) | 106.3±23.0 | 89.4±7.1 | **<0.0001** |
| Montreal Cognitive Assessment Adjusted Total Score | 25.6±2.2 | 27.0±5.4 | 0.185 |
| Montreal Cognitive Assessment Raw Total Score | 25.5±2.2 | 28.0±2.0 | **<0.0001** |
| Digit Symbol Substitution Task Score | 55.2±12.2 | 84.9±12.8 | **<0.0001** |
| Trailmaking A Time (seconds) | 36.8±11.1 | 23.8±8.4 | **<0.0001** |
| Trailmaking B Time (seconds) | 98.7±44.0 | 53.1±15.3 | **<0.0001** |
| fMRI – Volumes Removed Due to Motion | 7.9±13.9 | 8.4±9.8 | 0.813 |

Cohort mean values for key variables from analyses. Values following ‘±’ indicate the standard deviation of the variable in the cohort. Statistical comparison between cohorts was carried out using Welch’s T-tests. *For some measures, some participants did not have data available (Expanded Short Physical Performance Battery had 190 of 192 older adults with data available, Trailmaking B had 191 of 192 older adults with data available). In these cases, statistical comparison with the younger cohort was based on those older adults who had data for the measure.

| 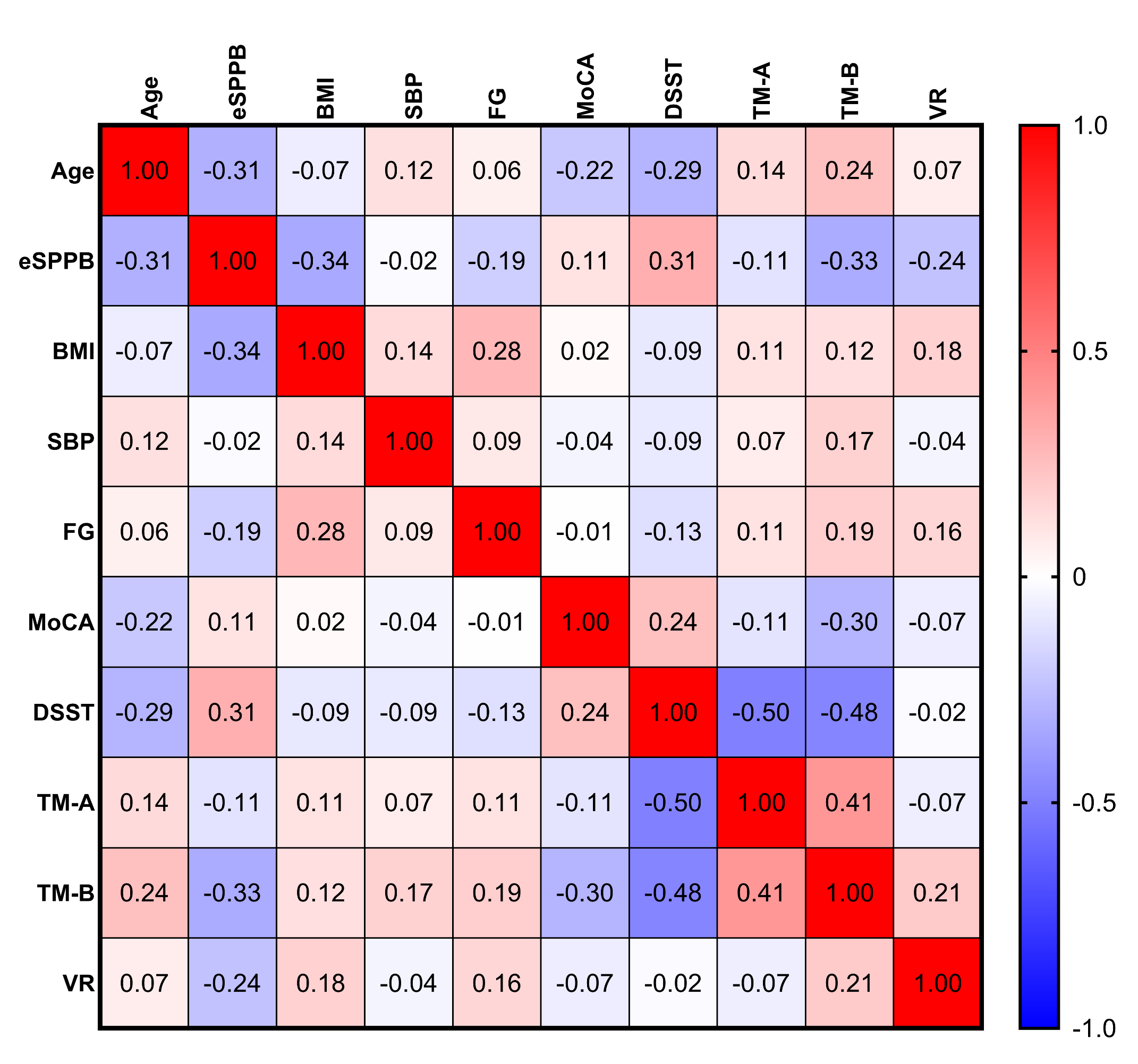  **Supplementary Figure 1.** Spearman correlation matrix for key variables in the cohort of older adults. Red indicates positive correlation while blue indicates negative correlation. As with Supplementary Table 1, correlations are based only on participants with data available. The variables follow the same order that is presented in Supplementary Table 1. Numeric values represent Spearman’s ρ from the relationship between variables.  *eSPPB –* Expanded Short Physical Performance Battery  *BMI* – Body Mass Index  *SBP* – Systolic Blood Pressure  *FG* – Fasting Blood Glucose  *MoCA* – Montreal Cognitive Assessment Raw Total Score  *DSST* – Digit Symbol Substitution Task Score  *TM-A* – Trailmaking A Time  *TM-B* – Trailmaking B Time  *VR* – Volumes Removed from resting-state fMRI scans due to motion |
| --- |

Supplementary Figure 1 shows the Spearman correlation coefficient between key variables in analyses and some additional variables of interest, such as performance on cognitive tests, for the sample of older adults. We note that some key variables of interest, such as eSPPB and BMI, were interrelated. These variables also shared a common association with in-scanner motion.

Supplementary Figure 2a shows results from a t-test of the entropy maps of younger and older adults with adjustment for the number of volumes removed from scans due to motion. Results did not meaningfully change from Figure 1 of the main text. Supplementary Figure 2b shows results from a regression of eSPPB against entropy maps while adjusting for in-scanner motion. Results were similar to Figure 2 from the main paper. The most noticeable difference was that the association in the basal ganglia was no longer significant following cluster correction for multiple comparisons, though the unthresholded image indicates that the effect estimate was only slightly weakened. Supplementary Figure 2c shows results from a regression of BMI against entropy maps while adjusting for in-scanner motion. Results were very similar to Figure 3 from the main paper.

| 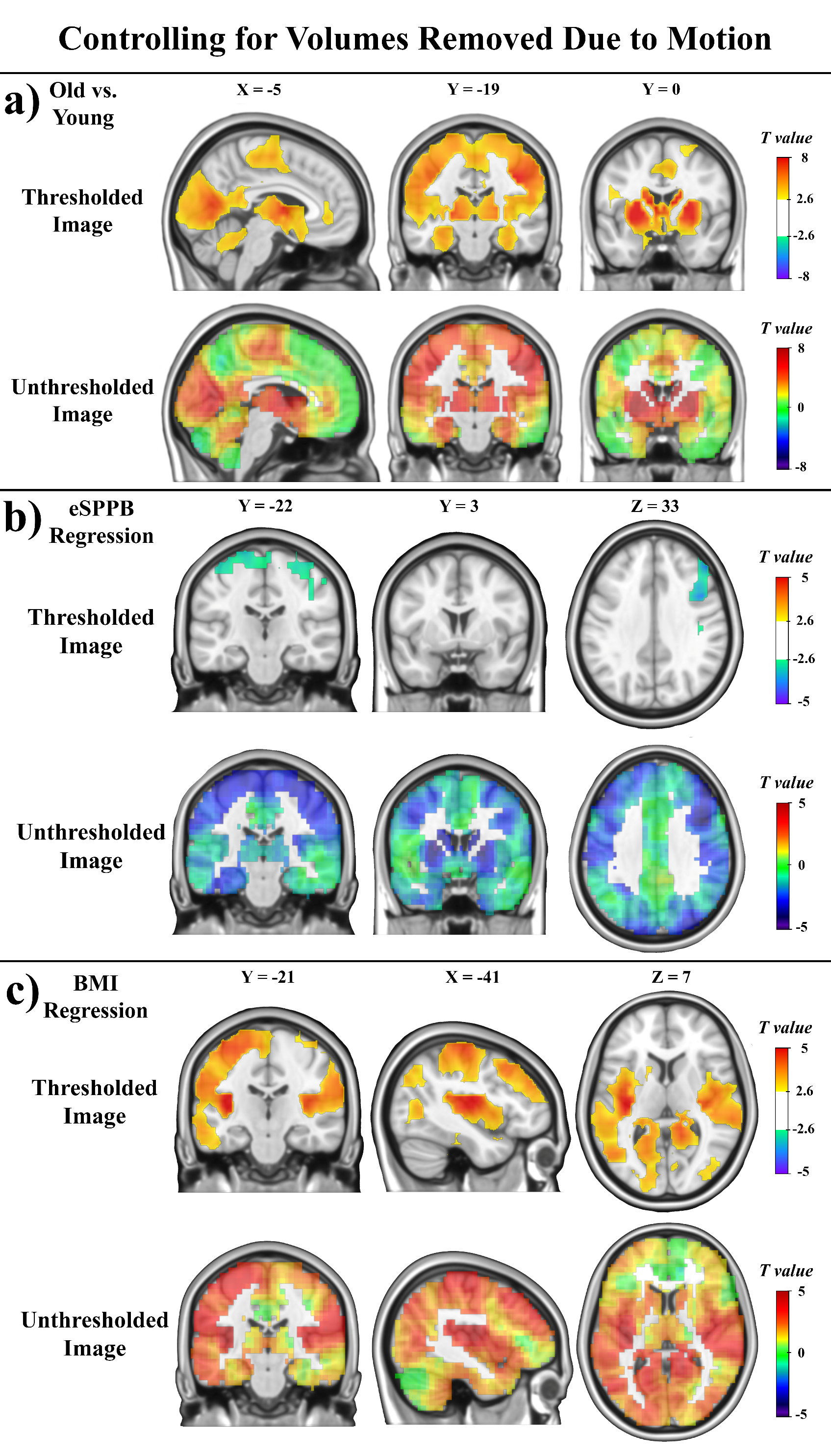  **Supplementary Figure 2.** Results from replication analyses controlling for the number of volumes removed due to motion. a) Thresholded and unthresholded maps of T-values from a two-sample t-test comparing entropy maps of a cohort of older adults and a cohort of younger adults while controlling for the number of volumes removed due to motion. Warmer colors indicate regions with higher entropy in the older adults. b) Thresholded and unthresholded maps of T-values from a regression of eSPPB against brain entropy maps in the cohort of older adults while controlling for the number of volumes removed due to motion. Cooler colors indicate a negative association between eSPPB and entropy. c) Thresholded and unthresholded maps of T-values from a regression of BMI against brain entropy maps in the cohort of older adults while controlling for the number of volumes removed due to motion. Warmer colors indicate a positive association between BMI and entropy. |
| --- |

Supplementary Figure 3 shows results from a regression of the interaction of age group (older = “1”, younger = “0”) and BMI against entropy maps. There were no significant associations that survived correction for multiple comparisons. This indicates that the relationship between BMI and brain entropy did not significantly differ between the older and younger age groups in any brain regions.

| 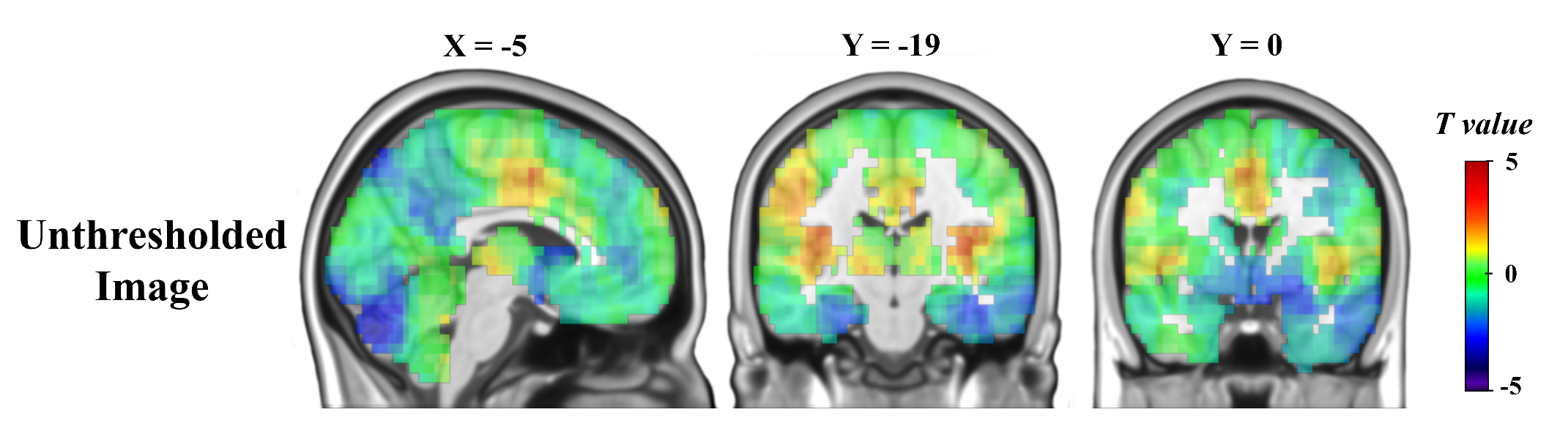  **Supplementary Figure 3.** Unthresholded map of T-values from a regression of the interaction between age group and BMI against brain entropy maps. Warmer colors indicate a positive association between the interaction term and entropy while cooler colors indicate a negative relationship. There were no significant clusters following cluster correction for multiple comparisons. |
| --- |

Given the strength of the difference between the older and younger cohorts (Figure 1), we evaluated whether age was associated with entropy within the older sample. A regression model was fit in the older adult sample with age as the sole regressor and entropy maps as the outcome. Supplementary Figure 4 shows results from this regression. No clusters remained significant following correction for multiple comparisons. Therefore, only unthresholded images are shown. The regions that exhibited the largest (though not statistically significant) associations between age and entropy were located in the dorsal parietal, occipital, and medial prefrontal cortex, as well as in the basal ganglia.

| 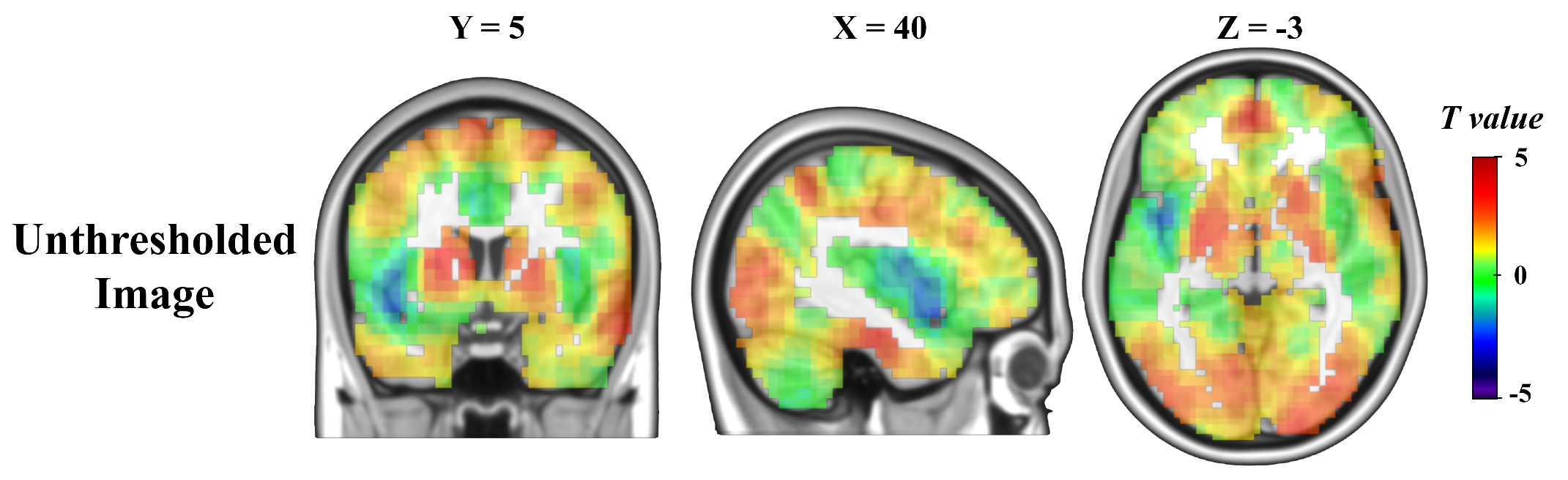  **Supplementary Figure 4.** Unthresholded maps of T-values from a regression of age against brain entropy maps in the cohort of older adults. Warmer colors indicate a positive association between eSPPB and entropy while cooler colors indicate a negative relationship. There were no significant clusters following cluster correction for multiple comparisons. |
| --- |

In further exploratory analyses, we evaluated several other measures of general health. Results from these exploratory analyses are shown in Supplementary Figures 5-8.

Supplementary Figure 5 shows results from a T-test of older participants with and without type 2 diabetes history. Participants with type 2 diabetes had significantly higher entropy in a cluster of voxels spanning from the midcingulate to the left sensorimotor cortex.

| 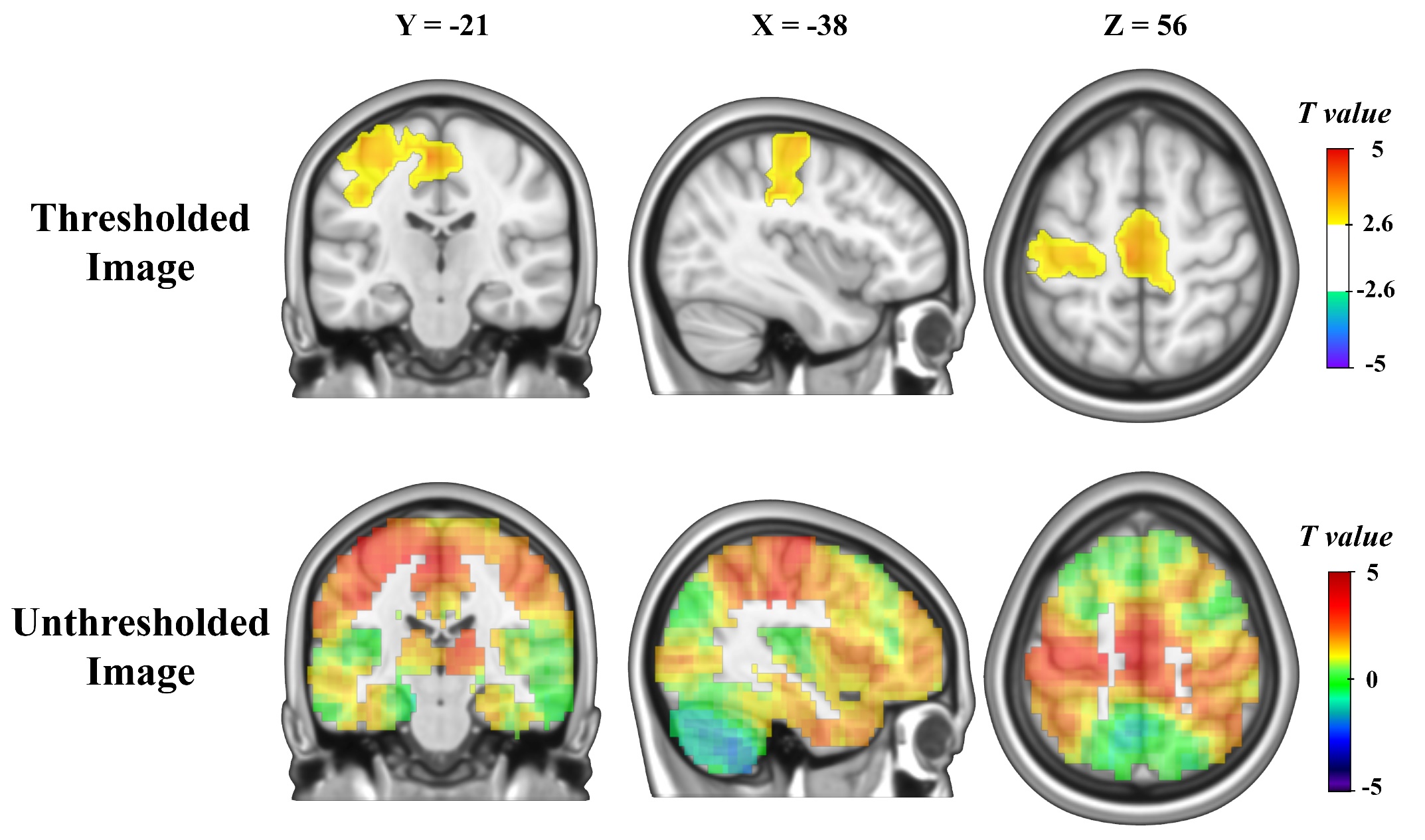  **Supplementary Figure 5.** Thresholded and unthresholded maps of T-values from a two-sample t-test comparing entropy maps of older adults with and without diabetes history. Warmer colors indicate regions with higher entropy in older adults with diabetes history. |
| --- |

Supplementary Figure 6 shows results from a regression of fasting blood glucose against entropy maps. Higher fasting glucose was associated with higher entropy in portions of the left insula and temporal lobe.

| 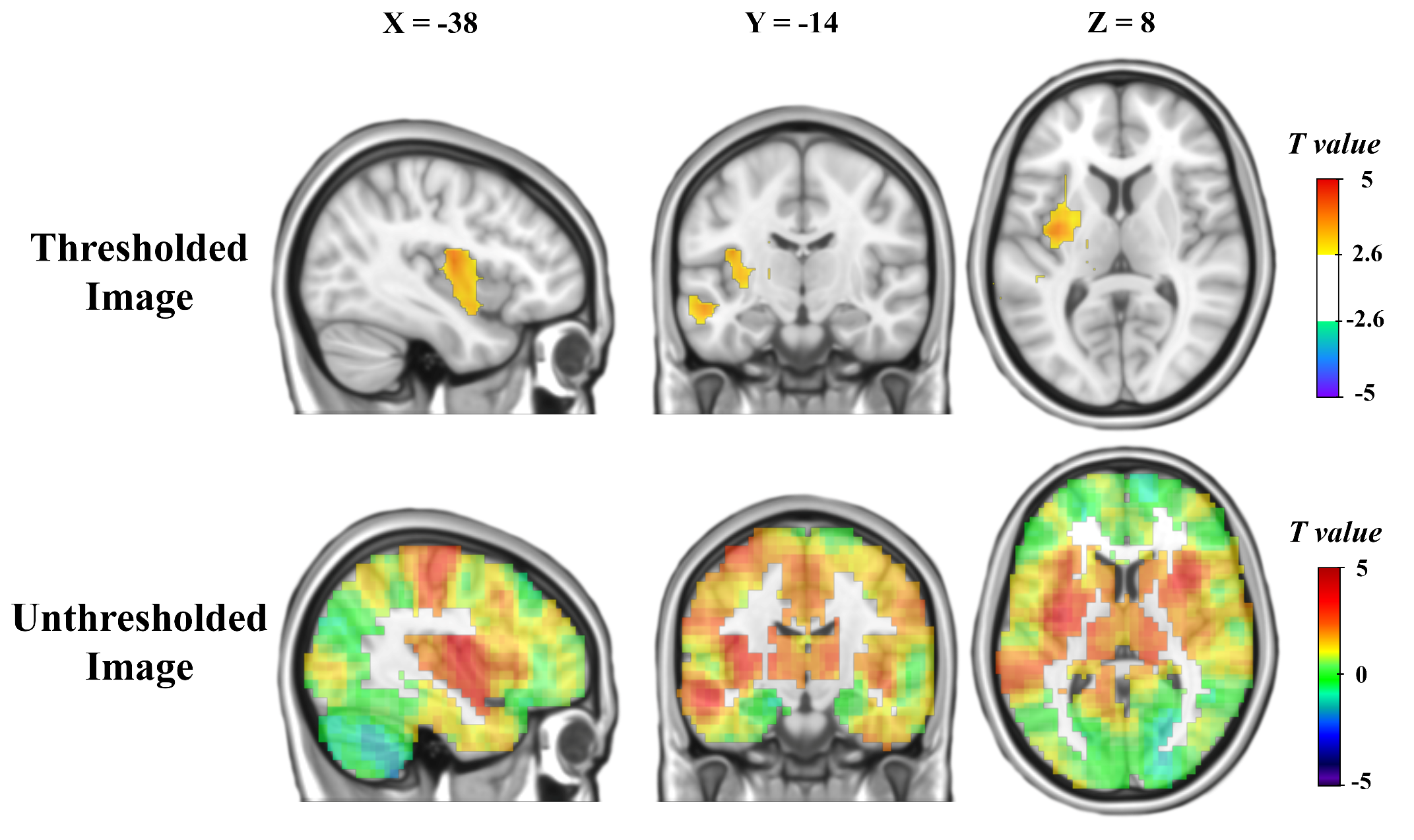  **Supplementary Figure 6.** Thresholded and unthresholded maps of T-values from a regression of fasting blood glucose against brain entropy maps in the cohort of older adults. Warmer colors indicate a positive association between fasting glucose and entropy while cooler colors indicate a negative relationship. |
| --- |

Supplementary Figure 7 shows results from a T-test of older participants with and without hypertension. Participants with hypertension had significantly higher entropy in the majority of the occipital lobe.

| 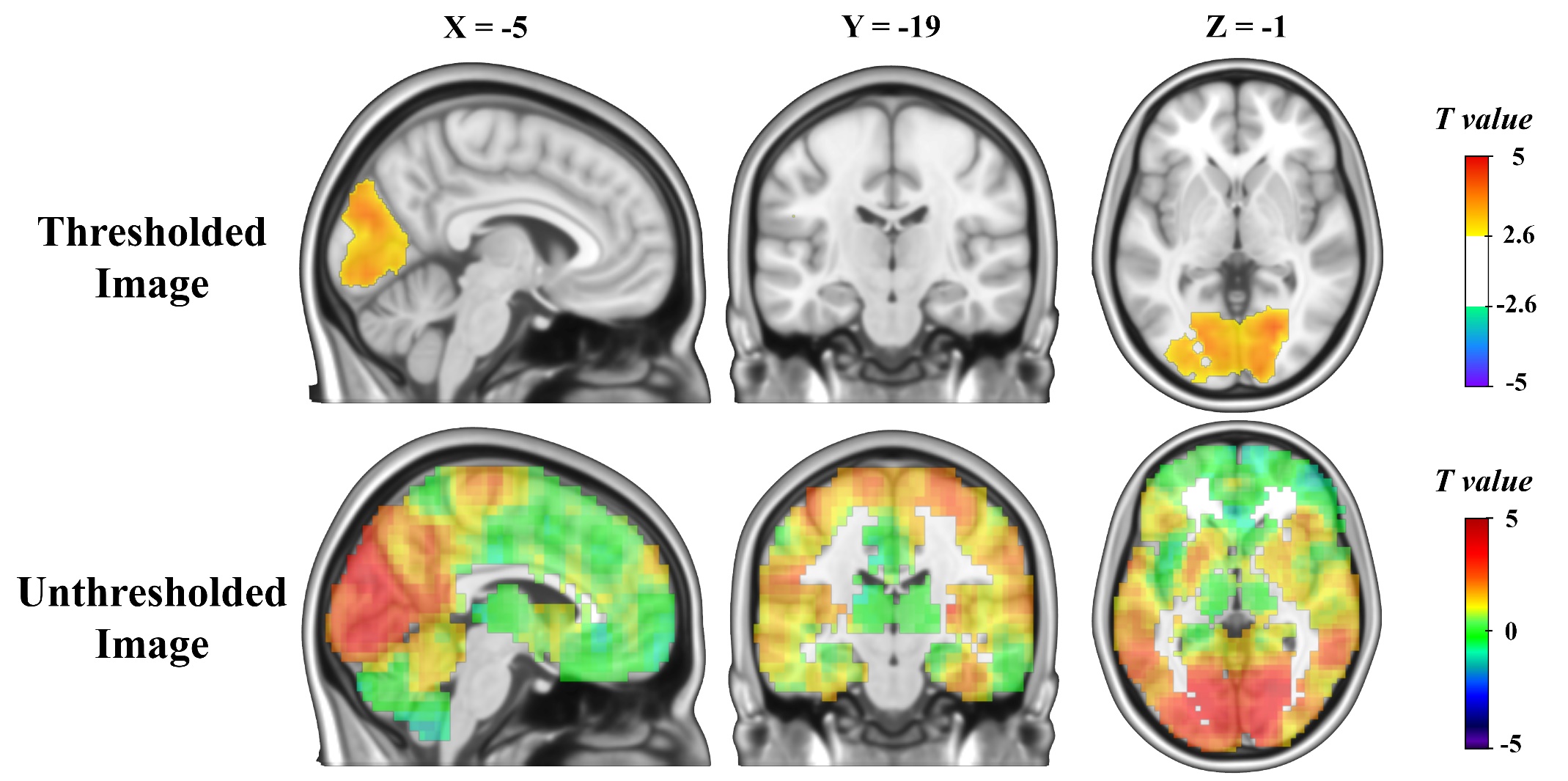  **Supplementary Figure 7.** Thresholded and unthresholded maps of T-values from a two-sample t-test comparing entropy maps of older adults with and without hypertension history. Warmer colors indicate regions with higher entropy in older adults with hypertension. |
| --- |

Supplementary Figure 8 shows results from a regression of systolic blood pressure against entropy maps. Systolic blood pressure was associated with higher entropy in portions of the occipital and parietal lobes.

| 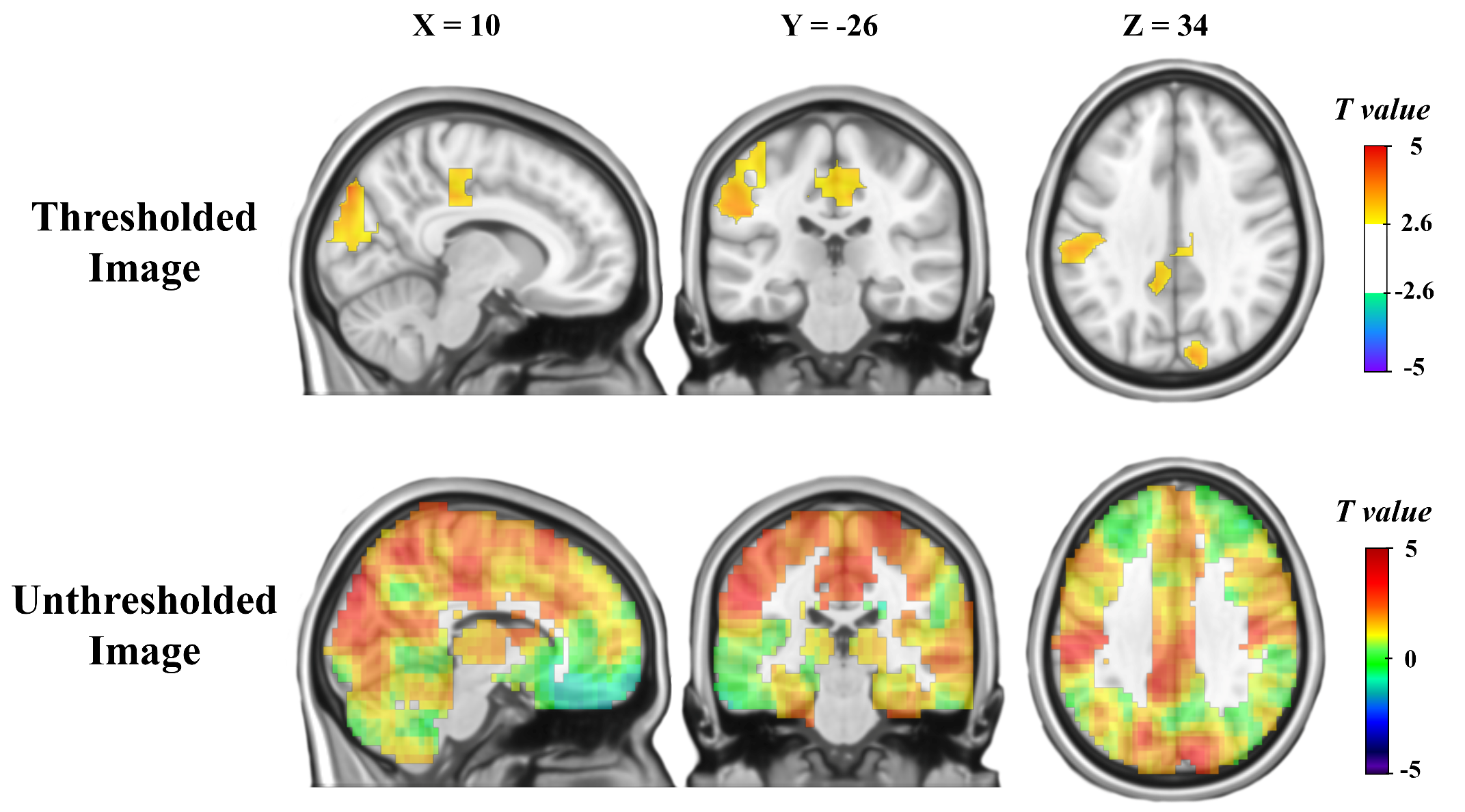  **Supplementary Figure 8.** Thresholded and unthresholded maps of T-values from a regression of systolic blood pressure against brain entropy maps in the cohort of older adults. Warmer colors indicate a positive association between systolic blood pressure and entropy while cooler colors indicate a negative relationship. |
| --- |

Given the significant relationships between entropy and fasting glucose and systolic blood pressure and the fact that both fasting glucose and systolic blood pressure were significantly higher in older adults compared to younger adults, we assessed if findings from the main paper (i.e., those in Figures 2-4) differed when controlling for fasting glucose and systolic blood pressure. In Supplementary Figure 9a, the results from a T-test of entropy maps between older and younger adults (while controlling for differences in fasting glucose and systolic blood pressure) is shown. The effects are slightly weakened but the interpretation of the finding remains unchanged from the result reported in the main text. In Supplementary Figure 9b and 9c, the association with brain entropy is assessed for eSPPB score and BMI, respectively, while controlling for fasting glucose and systolic blood pressure. Again, the effect sizes are slightly weakened compared to models that do not control for fasting glucose ad systolic blood pressure, but most clusters of voxels that were significant without controlling for these factors remain significant and the overall interpretation of the images remains unchanged. The most noticeable change is that when controlling for these additional factors, a cluster in the left basal ganglia is no longer significantly related to eSPPB score. The unthresholded images clearly remain very similar to the corresponding images in the main text.

| 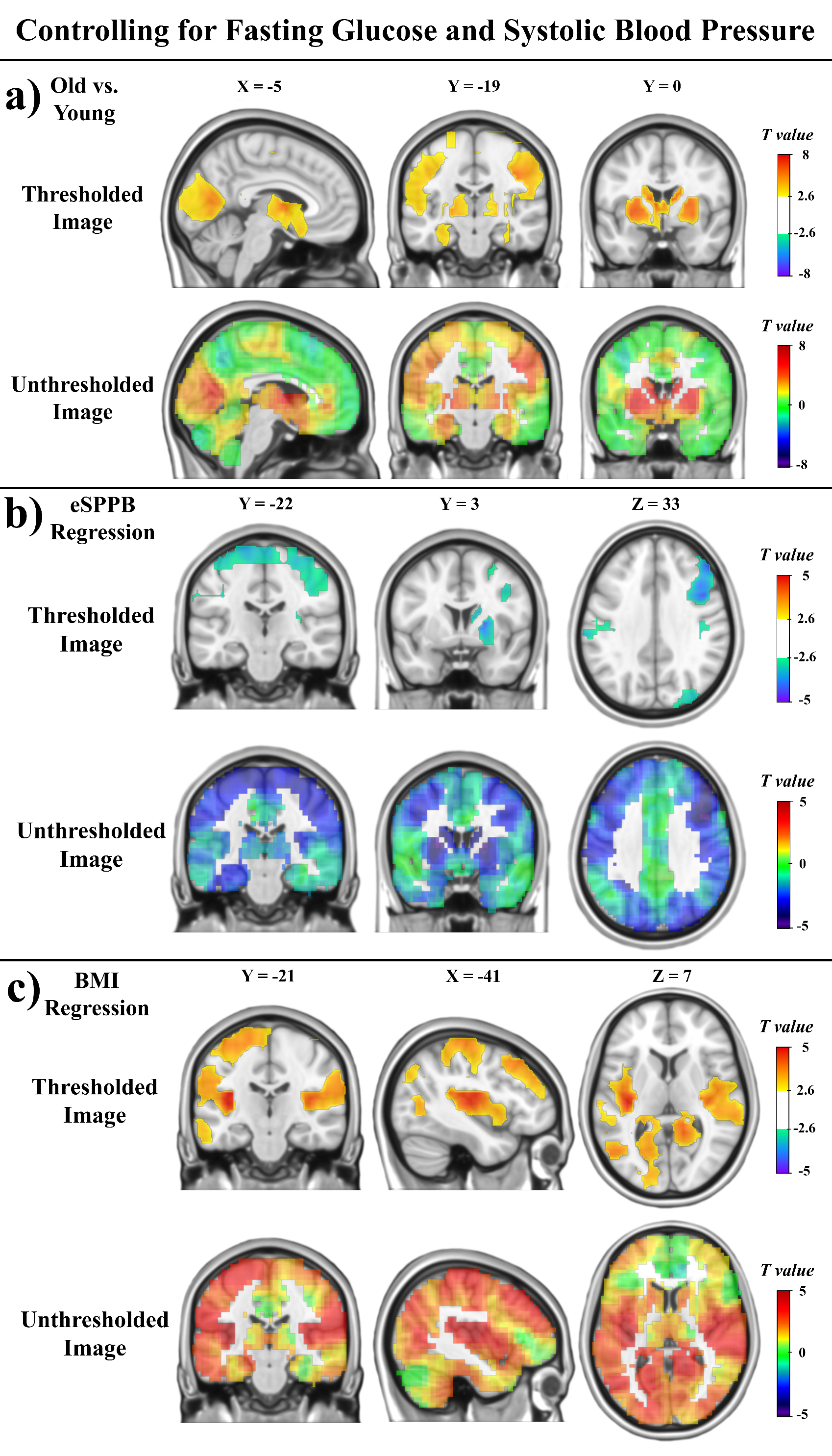  **Supplementary Figure 9.** Results from replication analyses controlling for fasting glucose and systolic blood pressure. a) Thresholded and unthresholded maps of T-values from a two-sample t-test comparing entropy maps of older adults and younger adults while controlling for fasting glucose and systolic blood pressure. Warmer colors indicate regions with higher entropy in older adults with diabetes history. b) Thresholded and unthresholded maps of T-values from a regression of eSPPB against brain entropy maps in the cohort of older adults while controlling for fasting glucose and systolic blood pressure. Cooler colors indicate a negative association between eSPPB and entropy. c) Thresholded and unthresholded maps of T-values from a regression of BMI against brain entropy maps in the cohort of older adults while controlling for fasting glucose and systolic blood pressure. Warmer colors indicate a positive association between BMI and entropy. |
| --- |

Supplementary Figure 10 shows results from a two-sample t-test of older participants who did or did not report exercising at moderate intensity for at least 150 minutes per week. Physically active individuals had significantly lower entropy in the basal ganglia and portions of the left and right hippocampus.

| 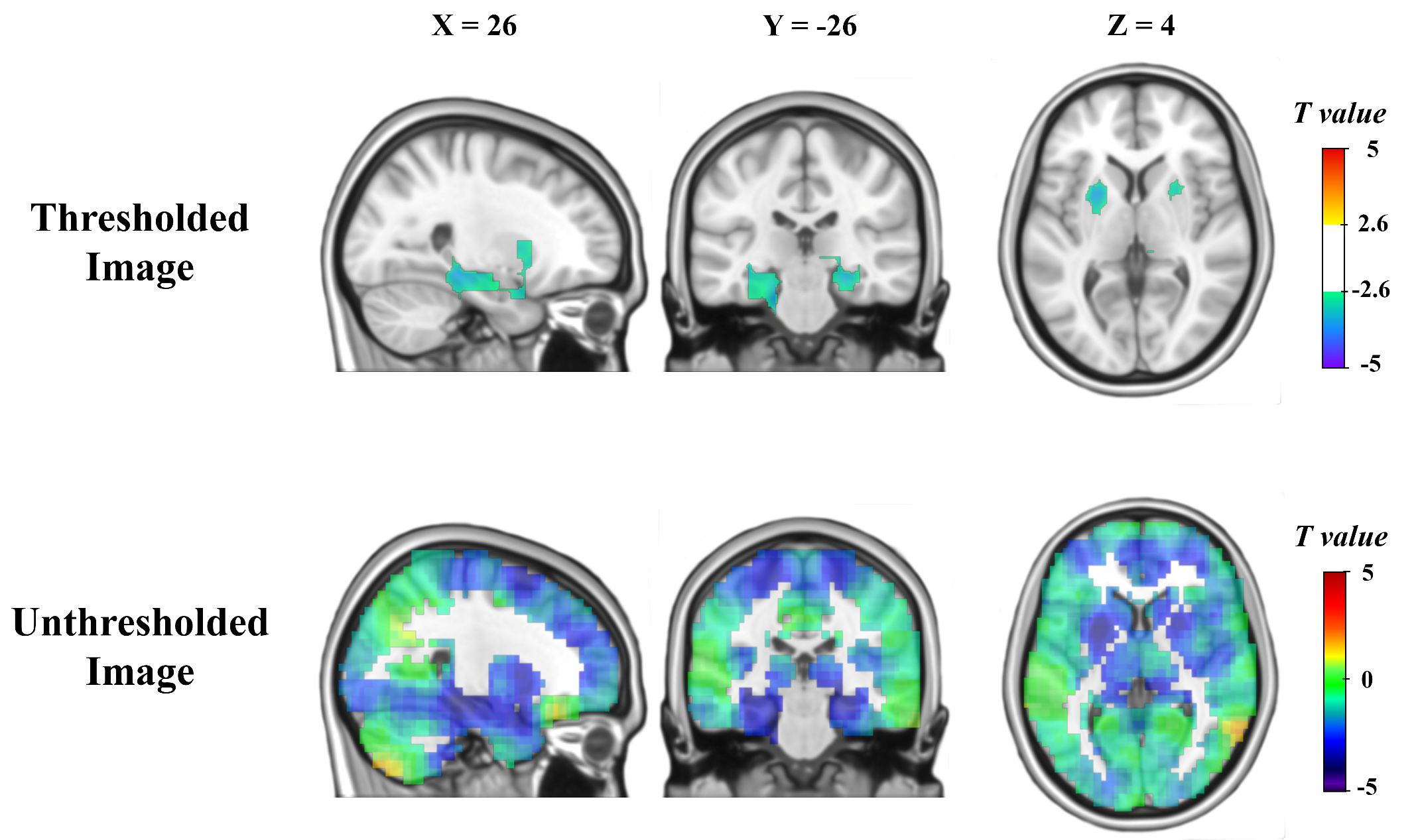  **Supplementary Figure 10.** Thresholded and unthresholded maps of T-values from a two-sample t-test comparing entropy maps of older adults who do meet physical activity guidelines versus older adults who do not. Cooler colors indicate regions with higher entropy in the physically inactive older adults. |
| --- |

Supplementary Figure 11 shows results from replication analyses using the Leiden community detection algorithm. We found that older participants still had higher entropy than younger participants (Supplementary Figure 11a), that higher eSPPB was still associated with lower entropy in regions including the sensorimotor cortex, basal ganglia, and frontal lobe (Supplementary Figure 11b), and that higher BMI was still associated with higher entropy in widespread cortical regions (Supplementary 11c).

| 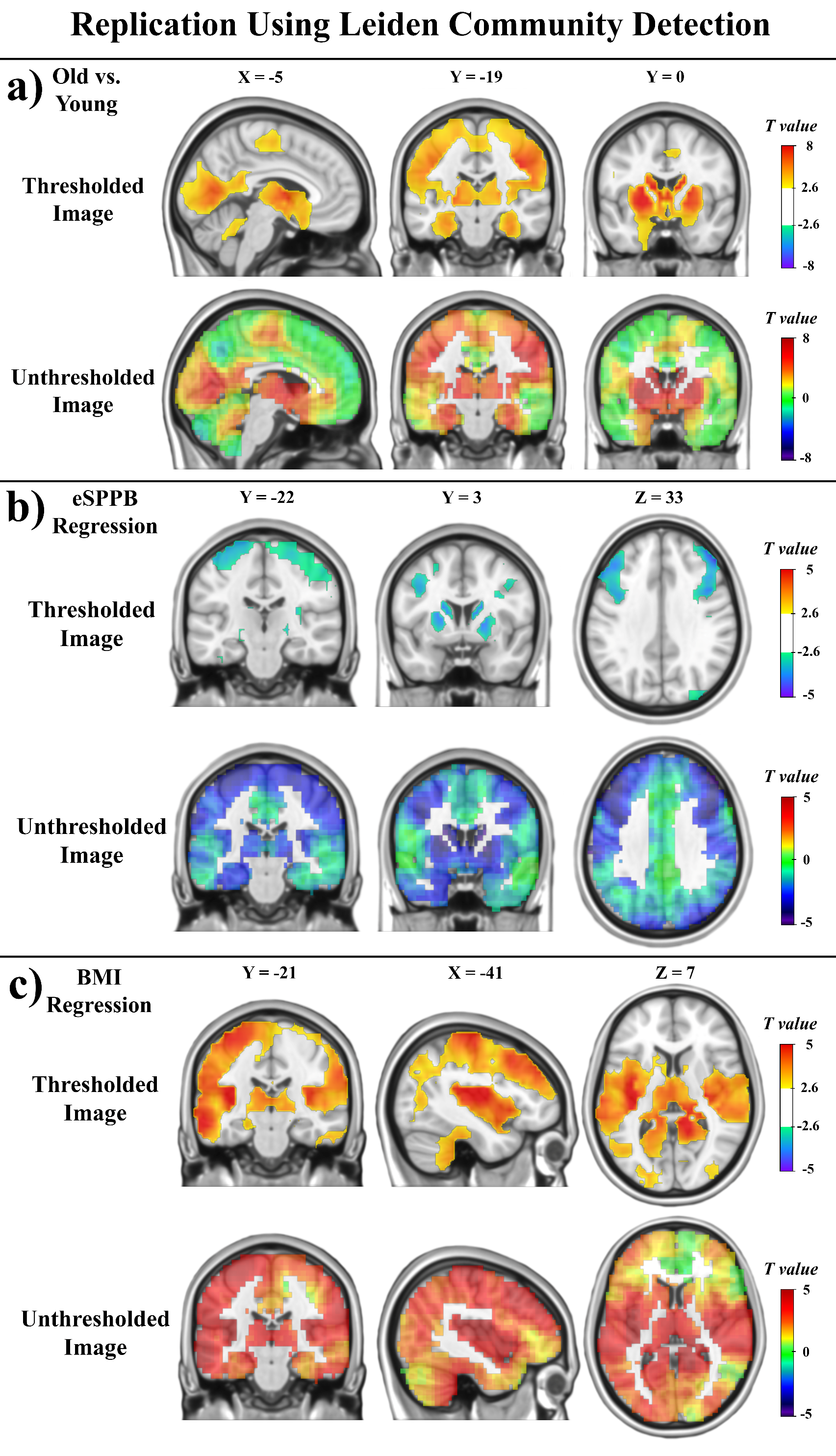  **Supplementary Figure 11.** Results from replication analyses using communities identified with the Leiden algorithm. a) Thresholded and unthresholded T-value maps from a t-test comparing entropy maps of older and younger adults. Warmer colors indicate higher entropy in the older adults. b) Thresholded and unthresholded T-value maps from a regression of eSPPB scores against entropy maps in older adults. Cooler colors indicate regions where higher entropy is associated with worse physical function. c) Thresholded and unthresholded T-value maps from a regression of BMI scores against entropy maps in older adults. Warmer colors indicate regions where higher entropy is associated with higher BMI. |
| --- |

Supplementary Figure 12 shows results from analyses assessing the relationship between gray matter volume and entropy as well as analyses replicating findings from the original paper while controlling for gray matter volume. Results indicated that having more gray matter is associated with higher entropy in regions including the precuneus, medial prefrontal cortex, lateral parietal lobe, and right inferior frontal gyrus (Supplementary Figure 12a). Notably, there was little to no relationship between gray matter volume and entropy in the sensorimotor cortex, occipital lobe, or basal ganglia, which were the key areas in analyses from the main paper. Including gray matter volume for each voxel as a covariate in a regression with entropy as the dependent variable and age group as the independent variable of interest revealed results that were highly similar to those shown in Figure 2 of the main paper (Supplementary Figure 12b). Similarly, including gray matter volume for each voxel as a covariate in regressions with entropy as the dependent outcome and eSPPB as the independent variable of interest revealed results that were highly similar to results in Figure 3 of the main paper (Supplementary Figure 12c). Finally, including gray matter volume for each voxel as a covariate in regressions with entropy as the dependent outcome and BMI as the independent variable of interest revealed results that were highly similar to results in Figure 4 of the main paper (Supplementary Figure 12d).

| 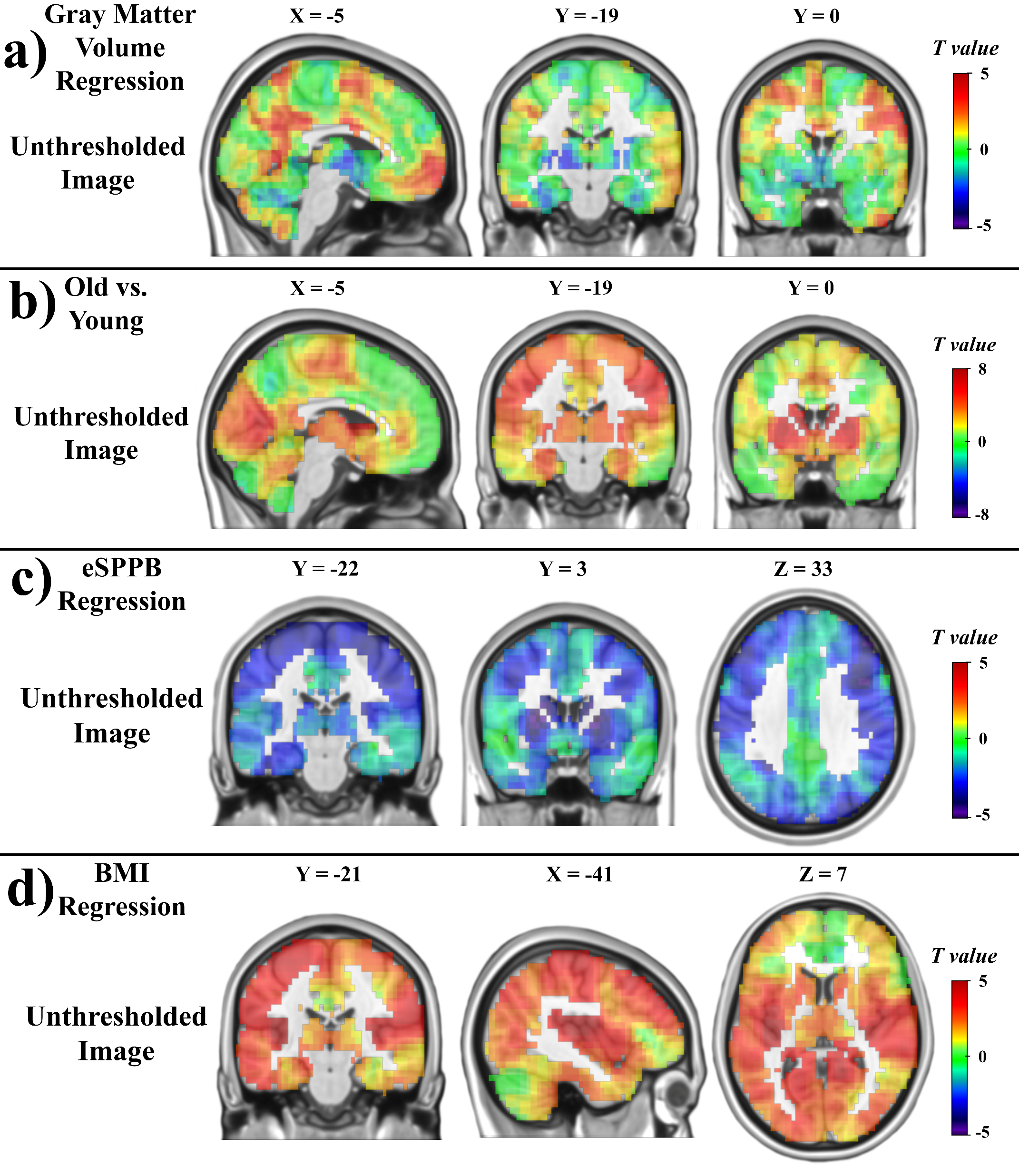**Supplementary Figure 12.** Results from analyses of the relationship between gray matter volume and entropy and the effect of including gray matter volume as a covariate in regressions from the main analyses of this paper. a) Unthresholded T-value maps from a regression comparing entropy maps to gray matter volume maps in older adults. Warmer colors indicate regions where more gray matter is associated with higher entropy. b) Unthresholded T-value maps from a regression comparing entropy maps of older and younger adults while controlling for differences in gray matter volume. Warmer colors indicate higher entropy in the older adults. c) Unthresholded T-value maps from a regression of eSPPB scores against entropy maps in older adults while controlling for differences in gray matter volume. Cooler colors indicate regions where higher entropy is associated with worse physical function. d) Unthresholded T-value maps from a regression of BMI scores against entropy maps in older adults while controlling for differences in gray matter volume. Warmer colors indicate regions where higher entropy is associated with higher BMI. |
| --- |

Stewart, A. L., Mills, K. M., King, A. C., Haskell, W. L., Gillis, D., & Ritter, P. L. (2001). CHAMPS physical activity questionnaire for older adults: outcomes for interventions. *Med Sci Sports Exerc*, *33*(7), 1126-1141. <https://doi.org/10.1097/00005768-200107000-00010>
