# Supplementary material for: Poorer Physical Function Is Associated With Elevated Spatial Entropy in the Aging Brain Network Landscape
Source: Aging Cell. 2026 Jun 18;25(6):e70596. doi: 10.1111/acel.70596 (PMC13277748; doi:10.1111/acel.70596)
Supplement: Supplementary file 2 — Data S2. Aging cell author checklist. [file ACEL-25-e70596-s001.docx]

**AGING CELL AUTHOR CHECKLIST**. ***Authors should submit this checklist together with their manuscript. Please ensure that you have read the Author Guidelines in detail before submission.***

| **Title** | Poorer physical function is associated with elevated spatial entropy in the aging brain network landscape | | | | | | |
| --- | --- | --- | --- | --- | --- | --- | --- |
| **Authors** | Clayton C. McIntyre, Shannon M. O’Donnell, Robert G. Lyday, Jonathan H. Burdette, Steven R. Cummings, Stephen B. Kritchevsky, Paul J. Laurienti | | | | | | |
| **Manuscript Type** | Research Article | | | | | | |
| **Total Character Count (including spaces)^1^** | 35,175 | | | | | | |
| **Word count of Summary^2^** | 177 | | | | | | |
| **Number of papers cited in the References^3^** | 64 | | | | | | |
| **Listing of all Tables (Table1, Table 2 etc)^4^** | NA | | | | | | |
|  |  | | | | | | |
| **Figure specifications (please complete one row per figure)^5^**  ***Figure no.*** | Colour  ***(yes/no)*** | Greyscale  ***(yes/no)*** | Black and white  ***(yes/no)*** | Single column (80mm)  ***(yes/no)*** | Double column (180mm)  ***(yes/no)*** | Size of figure at full scale  (mm x mm)  ***(insert details)*** | Smallest font size used in the figure at full scale (minimum 6pt)  ***(insert***  ***details)*** |
| 1 | Yes | No | No | No | Yes | 616.3 x 374.6 | 24 |
| 2 | Yes | No | No | No | Yes | 514.8 x 255.5 | 24 |
| 3 | Yes | No | No | No | Yes | 489.4 x 279.7 | 24 |
| 4 | Yes | No | No | No | Yes | 489.4 x 288.9 | 24 |
| 5 | Yes | No | No | No | Yes | 557.5 x 298.5 | 24 |

**^1^** The maximum character count allowed is 50,000 (incl. spaces) for Primary Research Papers and Reviews, 10,000 for Short Takes.

**^2^** Summary should not exceed 250 words.

**^3^** Primary Research Papers can contain a maximum of two tables. If more are needed they should replace some of the Figures or can be placed in the Supporting Information.

**^4^** A maximum of 45 references is allowed for Primary Research Papers and 20 references for Short Takes.

**^5^** A Primary Research Paper may contain up to 6 figures and a Short Take up to 2 figures. Authors are encouraged to provide figures in the size they are to appear in the journal and at the specifications given.
